# Contemporary Concepts of Adhesive Cementation of Glass-Fiber Posts: A Narrative Review

**DOI:** 10.3390/jcm13123479

**Published:** 2024-06-14

**Authors:** Panayiotis Tsolomitis, Sofia Diamantopoulou, Efstratios Papazoglou

**Affiliations:** Department of Operative Dentistry, Dental School, National and Kapodistrian University of Athens, 11527 Athens, Greece; petetsolomitis@gmail.com (P.T.); sophia.diamantopoulou@gmail.com (S.D.)

**Keywords:** glass fiber posts, fiberglass posts, cementation technique, post length, resin cement, adhesive bonding, bond strength, surface pretreatment, endodontic sealer, root canal irrigant

## Abstract

(1) Background: Cementation of glass fiber posts to root canals has been associated with various failures, especially debonding. This narrative review aims to present the contemporary concepts concerning the adhesive cementation of glass fiber post and to discuss the optimal management of these factors. (2) Methods: Electronic search was performed in MEDLINE/Pub Med and Google Scholar using selected keywords examining the parameters post length, surface treatment of glass fiber posts, post space preparation and dentin pretreatment, resin cement selection, adhesive systems and hybrid layer formation, and clinical techniques. (3) Results: The search led to the selection of 44 articles. Epoxy resin-based endodontic sealers are recommended and the use of temporary cement in the root canal should be avoided. The minimum length of a glass fiber post adhesively cemented to a root canal is 5 mm. Irrigating the root canals with chlorhexidine, MTAD, or EDTA (alone or in combination with NaOCl) after post space preparation seems to enhance the bond strength. Silane application on the surface of the post seems to be beneficial. Concerning resin cements and adhesive systems, the results were rather inconclusive. Finally, resin cement should be applied inside the root canal with an elongation tip and photoactivation should be delayed. (4) Conclusions: Contemporary concepts of adhesive cementation of glass fiber posts can indeed improve the bond between glass fiber posts, resin cement, and root canal dentin, however, evidence coming from long-term randomized prospective clinical trials is needed in order to obtain safer conclusions.

## 1. Introduction

Root canal posts are primarily used for retaining the coronal restorative material of endodontically treated teeth. The type of tooth and the quantity of remnant coronal tooth structure are important considerations for deciding if a post is necessary. Post placement is recommended for root-treated teeth with no remaining coronal walls [1] and those donewith one remaining wall [2]. Several types of post and core systems have been used so far. The decision for the appropriate post is challenging and depends mostly on the amount and the quality of the remaining tooth substance [3].

Prefabricated glass fiber posts have been used for a long time for the restoration of endodontically treated teeth. They are composed of glass fibers embedded in an epoxy resin-based or methacrylate-based matrix. One of their primary advantages is esthetics. Moreover, due to their similar elastic modulus with dentin and luting material, they show more uniform stress distribution and reduced risk of root fractures when compared with more rigid post materials [4,5]. Adhesive cementation of glass fiber posts has been demonstrated to have many advantages. Glass fiber posts (GFPs) that are adhesively bonded to the root canal can better withstand functional forces and have better retention.

Debonding has been identified as one of the most common type of failures for teeth restored with glass fiber posts [6]. Debonding is associated with problems in the adhesive bonding between the glass fiber post, the resin cement system, and the root canal dentin [6]. Numerous factors affect this adhesive bonding [6,7], including the configuration of the root canal, challenges in reaching the middle and apical root thirds, variations in the root dentin’s histology (number and orientation of dentinal tubules), and challenges in light curing in the middle and apical thirds [8,9]. Furthermore, the polymerization shrinkage stress creates gaps in the interfaces and lowers the adhesive bonding strength [10]. The effect of shrinkage stress within the root canal is elevated by the geometric configuration of the root canal (C-factor) [11]. Because the adhesive strength in this zone is weaker than the bond strength to the resin cement/GFP interface, gaps typically form at the dentin/resin cement interface. Contemporary concepts of cementation of glass fiber posts focus on improving the bond between glass fiber posts, resin cement, and root canal dentin, thus improving the longevity of restorations. The main factors in this field include the surface treatment of glass fiber posts, post space preparation and dentin pretreatment, resin cements, adhesive systems and hybrid layer formation, and clinical techniques.

This narrative review aims to present the contemporary concepts concerning the adhesive bonding of glass fiber posts and to discuss the optimal management of these factors before and during cementation procedure in order to achieve optimal adhesive bonding of the glass fiber post to the root dentin.

## 2. Materials and Methods

### 2.1. Data Sources

An electronic search was performed in MEDLINE/Pub Med and Google Scholar using the following keywords: glass fiber posts, fiberglass posts, cementation technique, post length, resin cement, adhesive bonding, adhesive cementation, bond strength, surface pretreatment, endodontic sealer, and root canal irrigants. Furthermore, to obtain more data during the review process, we extended our search by reviewing the reference sections of some of the retrieved articles.

### 2.2. Eligibility Criteria

The literature search was performed according to the following eligibility criteria: (1) written in the English language, (2) publication date between 2014 and 2024, (3) evaluation of factors affecting glass fiber post adhesive cementation, and (4) use of human or bovine teeth. The exclusion criteria were as follows: (1) use of artificial teeth, (2) use of primary or not fully formed teeth, (3) evaluation of adhesion only between glass fiber post’s surface and resin cement, (4) evaluation of post systems other than glass fiber, and (5) comparison between different post systems.

### 2.3. Search Strategy

**Figure 1:**
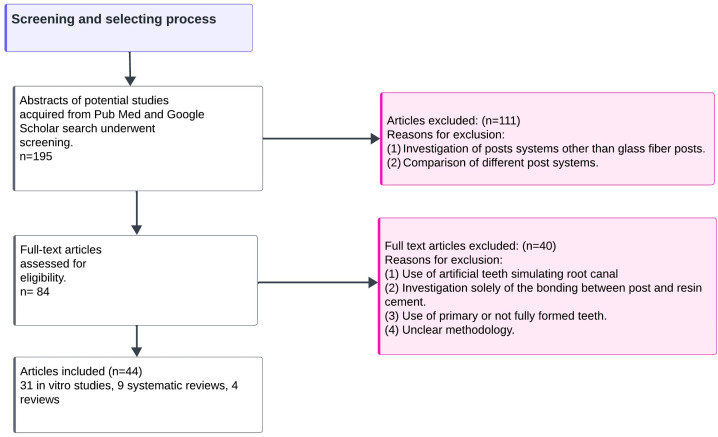
Flow diagram of the screening and selection process.

After eliminating duplicate papers, 195 abstracts were screened, and 84 articles focusing on the adhesive cementation of glass fiber posts were assessed for eligibility. After reading the full text articles, 40 of them were rejected because (1) they were conducted on artificial teeth stimulating root canals, (2) they evaluated adhesive bonding only between post surface and resin cement, (3) they were conducted on primary or not fully formed teeth, or (4) they used unclear methodologies (Figure 1).

### 2.4. Included Data 

Thus, in this narrative review, 31 in vitro studies were included. The selected studies are presented in Table 1. The conclusions from two reviews, eight systematic reviews, and one integrative review are also briefly presented.

## 3. Results

The findings from all articles were categorized according the following main factors affecting adhesive cementation of the post to root canal: endodontic sealers used, post length, root canal irrigants, surface pretreatment of glass fiber posts, resin cement, and cementation technique. Each of these will be further analyzed.

### 3.1. Endodontic Sealers

Various types of endodontic sealers have been used as part of root canal treatment. However, adhesive cementation of the post may be affected by the endodontic sealer used for root canal filling. Eugenol-based sealers must be avoided [7,13]. Eugenol-based sealers affect the adhesive bonding between resin cement and root canal dentin, regardless of the type of resin cement used. Eugenol’s hydroxyl groups are bonded to the monomer’s free radicals and decrease their reactivity. This reduces the degree of conversion of the resin cement and leads to a lower bond strength between the glass fiber post and the root canal [42]. However, it was suggested that it is possible to effectively remove eugenol-based sealer remnants using 95% alcohol or largo burs after preparing the post space [12]. Vilas-Boas et al. investigated three different endodontic sealers (a eugenol-based sealer, an epoxy-amine resin-based sealer, and a bioceramic sealer), and examined two different moments of cementation (immediately or 7 days after canal obturation). The epoxy-amine-resin-based sealer exhibited the highest bond strength values, and the bond strength values between posts cemented immediately after or seven days after obturation did not differ dramatically, although the results indicated that is better to cement the post immediately after the obturation [13]. No difference was noticed between the epoxy-amine resin-based sealer group and the control group, in which no sealer was used, indicating that epoxy-amine-based sealers do not interfere with the adhesive bonding between resin cement and root canal dentin [13]. The lower bond strength in the bioceramic sealer group was attributed to the remnants of the sealer inside the dentinal tubules. Remnants of the bioceramic sealer are rich in calcium and phosphate, resulting in high alkalinity, and in a decrease in the effectiveness of etching with phosphoric acid [8]. Soares et al. tested five different endodontic sealers: a eugenol-based sealer, a salicylate resin-based sealer containing calcium hydroxide, an epoxy resin-based sealer, an epoxy resin-based sealer containing calcium hydroxide, and a calcium silicate-based sealer [14]. The epoxy resin-based sealer group exhibited the highest bond strength values, similar to the control group, where no sealer was used. The group with the eugenol-based sealer showed the lowest bond strength values. Thus, an epoxy resin-based sealer is the sealer of choice when a glass fiber post restoration is planned [14].

Zaniboni et al. investigated the effect of temporary cements on post cementation [15]. Cementation of temporary metallic posts with eugenol-based or eugenol-free cement had a negative effect on the bond strength between the glass fiber post and root canal regardless of the root third evaluated [15]. SEM of the root dentin surfaces revealed a high incidence of residues in the apical third of the post space, without significant differences among the temporary cements. Therefore, use of temporary cement in the root canal must be avoided [15].

### 3.2. Post Dimensions

Although post length is not directly related to the adhesive cementation of a glass fiber post, an interesting finding from the literature search was that a glass fiber post can be shorter than the 2/3 of the root length and that if they extend the post space more apically than a certain length, clinicians do not favor adhesive bonding. The post length can be equal to 1/2 of the root canal length and not to 2/3 of the root length as previously believed [3]. Webber et al. evaluated glass fiber posts with different lengths and concluded that a post with a length equal to 1/2 of the root length is a viable solution when it is not possible to use a post with a length equal to 2/3 of the root length [16]. It has been suggested that the post length could be even shorter. Abdulrazzak et al. evaluated how the combination of ferrule height and post length affects the fracture resistance and failure mode of endodontically treated teeth restored with glass fiber posts, composite resin cores, and crowns [17]. They found significant differences among the different ferrule height groups but not among the different post length subgroups. Moreover, no significant interaction between ferrule height and post length was found. It should also be noted that most failures were restorable, except in the 0 mm ferrule and 5 mm post length subgroups [17]. Another study evaluated if the post length affects the fracture resistance of endodontically treated teeth. No significant differences were found between the three groups with different lengths of the glass fiber posts (5 mm, 7.5 mm, and 10 mm). It was therefore suggested that a post length over 5 mm did not have any impact on fracture resistance [18].

Freitas et al. investigated the effect of different glass fiber posts diameters on the push-out bond strength to dentin. The diameter of glass fiber posts had a significant effect on bond strength. Additionally, the glass fiber posts that were well adapted to the root canal presented higher bond strength values [19].

### 3.3. Root Canal Irrigants

After post space preparation, the clinician can use various root canal irrigants before proceeding to the cementation of the glass fiber posts [3]. A systematic review on this topic concluded that the combination of 17% EDTA and 2% sodium hypochlorite is the most effective combination, when resin cement with a self-etch adhesive system or self-adhesive resin cement is used [43]. Although ultrasonic activation of root canal irrigants can lead to more favorable results regarding debris removal and dentinal tubule opening, the use of the previously mentioned irrigants can lead to satisfactory results even without activation when self-adhesive resin cements are used [43]. Moreover, if an etch-and-rinse adhesive system is used, sodium hypochlorite 1% should be the root canal irrigant of choice [43].

Except for NaOCL/EDTA, the root canal can be additionally irrigated with chlorhexidine before glass fiber post cementation [20]. Durski et al. recommended a 2% chlorhexidine application for 60 s after performing irrigations with a combination of NaOCl/EDTA. In their study, the chlorhexidine treatment led to significantly better results regarding bond strength, regardless of the resin cement, root third, and thermocycling process [20]. Candido et al. investigated the effect of 2.5% NaOCl and 17% EDTA, 1% peracetic acid, 10% sodium ascorbate, and 20% alpha-tocopherol. In their study, the posts were cemented with a conventional resin cement in combination with an etch-and-rinse adhesive system. The use of the antioxidants after irrigation with NaOCl and EDTA increased the bond strength values, but when peracetic acid was used, the antioxidants had no effect [21].

Alkahtany evaluated four different root canal irrigants before glass fiber post cementation: EDTA, a mixture of doxycycline citric acid and detergent (MTAD), riboflavin, and pineapple peel extract. The irrigants were used in combination with NaOCl. Irrigations with MTAD and pineapple peel extract resulted in the highest mean bond strength values. In contrast, irrigations with EDTA or riboflavin displayed the lowest bond strength values with similar results between these two groups [22]. Jalali et al. used five different root canal irrigants before the cementation of glass fiber posts with a conventional resin cement combined with a self-etch adhesive system and concluded that etching or irrigating the root canals with MTAD or EDTA after post space preparation increases the bond strength between the resin cement and dentin [23].

Wan et al. investigated the effect of the MTAD solution and photon-induced photoacoustic streaming (PIPS) technique to remove the smear layer to increase the bond strength of adhesively cemented glass fiber posts. PIPS is a new type of laser agitation technique, where the laser is equipped with a special working head to avoid heat damage to the wall of the canal and periapical tissue. After an examination of the specimens with scanning electron microscopy, it was found that the combined effect of MTAD and the PIPS technique achieved the most complete smear layer removal, especially in the coronal third. Moreover, the combined effect of PIPS and MTAD led to the highest mean bond strength values. When MTAD or the PIPS technique was used alone, they led to only partially opened dentinal tubules [24].

### 3.4. Surface Pretreatment of Glass Fiber Posts

Different glass fiber post surface pretreatment methods have been tested to achieve better bonding between glass fiber posts and resin cement. The post surface can be modified by etching (by chemical or mechanical means) through silane application and the combination of both etching and silane application [7]. The main component of the matrix of most glass fiber posts is epoxy resin or methacrylate [6]. Most pretreatment methods aim to remove only the superficial resin layer while maintaining the integrity of the glass fibers [44]. Silane application alone cannot improve the retention of glass fiber posts to the root canal [44,45]. However, a surface modification method before the silane application leads to better results concerning the bond strength [46]. There are concerns about the use of air abrasion as a surface pretreatment method, since it does not only remove the superficial resin matrix but it could also possibly damage the glass fibers, jeopardizing post’s mechanical properties [44].

Elanghy et al. evaluated four different surface pretreatment methods. Bond strength values obtained with dichloromethane application for 10 min were significantly higher compared with those obtained with the other pretreatment methods. The application of 9% hydrofluoric acid resulted in significantly lower bond strength values compared to the control group (no surface pretreatment). Air abrasion led to significantly lower bond strength values in the coronal and the middle levels of the root compared with the control group. The application of silane did not enhance the bond strength [25]. They also examined post-surface changes after the four pretreatment methods using scanning electron microscopy. They noticed that the posts that did not undergo any surface pretreatment had a rather rough surface with some glass fibers exposed. Pretreatment with dichloromethane led to the dissolving of the resin matrix and the exposure of undamaged glass. On the other hand, glass fibers were damaged after pretreatment with air abrasion or hydrofluoric acid [25].

Samini et al. compared two surface pretreatment methods (9.5% hydrofluoric acid and 10% hydrogen peroxide) and evaluated if warmed silane leads to a higher bond strength. The results indicated that the warmed silane application significantly increased the bond strength. HF acid etching with warmed silane application led to the highest bond strength values [26]. On the other hand, Mishra et al. rejected the use of hydrofluoric acid due to its aggressiveness. They proposed surface etching with hydrogen peroxide for 60 s or phosphoric acid for 15 s. According to them, a longer application time may jeopardize the integrity of the glass fibers, whereas a longer application time of hydrogen peroxide may inhibit the polymerization of the resin cement due to the creation of an oxygen-rich surface [44].

Similarly, Majeti et al. evaluated surface treatment with phosphoric acid 37% or hydrogen peroxide 24% for 15, 30, and 60 s. Posts etched with phosphoric acid for 15 s and posts etched with hydrogen peroxide for 60 s had higher bond strength values with no significant difference between them. Scanning electron microscopy revealed that the two previous protocols achieved complete removal of the superficial epoxy resin layer without damaging the integrity of the fibers [27]. Rechia et al. noticed that warmed silane application or combination of silane with an adhesive system resulted in higher bond strength values compared with that acquired in a group where only silane was applied [28].

Santos et al. evaluated the influence of silane application, sandblasting, and etching with hydrogen peroxide. An increased bond strength was observed when silane was applied alone or in combination with hydrogen peroxide or sandblasting, with no significant difference among the groups receiving silanization [29]. They suggested that these results may be attributed to the inhibition of the polymerization of the resin cement by the hydrogen peroxide due to the formation of free radicals and a layer rich in oxygen by-products. On the other hand, sandblasting may have caused damage to the glass fibers [45]. Jana et al. used four different surface pretreatment methods: silane, air abrasion with silicon dioxide powder and silane, hydrofluoric acid etching for 10 s and silane, and silane combined with a universal bonding agent. They argued that there is a correlation between surface roughness and bond strength. Air abrasion followed by silane application resulted in the highest bond strength values. Moreover, they suggested that the use of a universal bonding agent is a clinically feasible alternative, and it helps increase the bond strength [30].

Gomez et al. evaluated the influence of post surface pretreatment with laser irradiation. They used three different types of lasers; Er:YaG, Er,Cr:YSGG, and 980 mm diode lasers. According to their findings, irradiation of the post surface with Er,Cr:YSGG significantly increased the bond strength [31].

Khalum et al. evaluated four different post-surface disinfection methods: autoclave sterilization, chlorhexidine, curcumin photosensitizer solution activated with blue LED curing light, and 35% phosphoric acid. The highest bond strength values were achieved in the groups where chlorhexidine or phosphoric acid was used. The bond strength values in the groups where autoclave sterilization or the curcumin photosensitizer solution was used were similar to those of the control group where no method of disinfection was used [32].

### 3.5. Resin Cements, Adhesive Systems, and Hybrid Layer Formation

Dual-cured resin cements are predominately used for glass fiber posts adhesive cementation. Conventional resin cement combined with etch-and-rinse or self-etch adhesive systems or self-adhesive resin cement can be used [6]. Puildo et al. compared a conventional resin cement with a self-adhesive resin cement. The polymerization shrinkage values did not differ, and they were higher at the cervical region of the root canal. Conventional resin cement displayed higher degree of conversion values. The highest bond strength values were achieved with the conventional resin cement at the cervical region, whereas the bond strength values were similar for both types of cement at the apical region [10]. Migliau et al. compared three different resin cements: a conventional resin cement in combination with an etch-and-rinse adhesive system, a conventional resin cement in combination with a self-etch adhesive system, and a self-adhesive resin cement. The highest bond strength values were achieved by the conventional resin cement combined with the etch-and-rinse adhesive system. They concluded that bond strength values are lower when the root canal is not etched with phosphoric acid before cementation [33]. Nadler et al. evaluated the performance of five different resin cements/adhesive systems. They used three conventional resin cements combined with etch-and-rinse adhesive systems, one conventional resin cement combined with a self-etch adhesive system, and one self-adhesive resin cement. The highest bond strength was observed in the groups with the conventional resin cements combined with etch-and-rinse adhesive systems. However, in the middle and the apical third, the group with the conventional resin cement combined with an etch-and-rinse adhesive system showed similar results to the group with the conventional resin cement combined with the self-etch adhesive system and the group with the self-adhesive resin cement. Scanning electron microscopy showed that conventional resin cements combined with etch-and-rinse adhesive systems created a more uniform hybrid layer with a greater number of resin tags compared to the hybrid layer created by the conventional resin cement combined with the self-etch adhesive system or the self-adhesive resin cement [34].

Yaman et al. also compared the bond strength values of glass fiber posts cemented either with a conventional resin cement combined with a self-etch adhesive system or with a self-adhesive resin cement. In their study, half of the specimens were subjected to a thermomechanical aging procedure (TMA). The conventional resin cement showed better results without the TMA procedure. On the other hand, the bond strength values of posts cemented with the self-adhesive resin cement were not affected by the TMA procedure and they were higher than those of the posts cemented with the conventional resin cement [35].

A systematic review conducted by Sakris-Onofre et al. and a systematic review conducted by Miotti et al. suggested that self-adhesive cement should be the resin cement of choice when it comes to glass fiber post cementation [8,47]. The results of a meta-analysis conducted by Anganon et al. are similar, suggesting that in the long term, self-adhesive resin cements may have better performance compared to conventional resin cements [48]. Self-adhesive resin cements are composed of acid monomers that promote demineralization of the tooth substrate and infiltrate the dentin, promoting micromechanical and chemical bonding of the resin cement to hydroxyapatite. The acid-based interaction between the resin cement and the dentin creates water and thus cement’s moisture tolerance is improved. Additionally, self-adhesive resin cements display lower polymerization stress compared to that of conventional resin cements and this may result in a higher bond strength when they are used [47]. When conventional resin cements combined with etch-and-rinse adhesive systems are used, accurate dentin moisture control and adequate infiltration of the adhesive solution into the tooth substrate are required. The use of self-etch adhesive systems is also complex as far as proper solvent evaporation and excess adhesive removal are concerned [47].

Some studies suggest that when conventional resin cements are used, it is better to combined them with self-etch adhesive systems [36]. Barcellos et al. used the same conventional resin cement combined with either an etch-and-rinse adhesive system or with a self-etch adhesive system. The bond strength values were significantly higher when the self-etch adhesive system was used [36]. Although a thicker hybrid layer was formed when the etch-and-rinse adhesive system was used, the quality of the hybrid layer had a greater impact on the bond strength values than its thickness itself [36]. The major difficulty when etch-and-rinse adhesive systems were used was achieving the optimal moisture inside the root canal [37]. Rezende et al. evaluated the effect of root canal dentin moisture on the bond strength and nanoleakage values. They concluded that a slight amount of moisture should be left inside the root canal to achieve the optimal results [37].

### 3.6. Cementation Technique

Irrespective of the resin cement/adhesive system used, the clinician must pay attention to some additional factors possibly affecting fiber post adhesive cementation. Regardless of the type of resin cement, it must be applied using an elongation tip to avoid voids and bubbles formation [38]. Durski et al. examined the bond strength of glass fiber posts that were cemented to root canals using two different cement application techniques (using an elongation tip or microbrush). Resin cement application with an elongation tip led to better results regarding bond strength at the apical third regardless of the resin cement used. Application with an elongation tip also led to better results regarding bond strength at the middle and cervical thirds only when a self-adhesive resin cement was used alone [38].

Photoactivation timing affects both the mechanical properties of the resin cement and the retention of glass fiber posts to the root canal [39]. Pereira et al. investigated how three different photoactivation timings (immediately after, after 3 min, and after 5 min) affects the mechanical properties of two conventional and one self-adhesive resin cement. They also evaluated how the photoactivation timing affects the bond strength values of the posts within each group. The 5 min delay significantly increased the bond strength values regardless of the resin cement used. They also implied that the resin cement’s mechanical properties indicate the degree of conversion. According to their study, the better mechanical properties of the self-adhesive resin cement when delayed photoactivation was applied may be attributed to the fact that delayed photoactivation did not prohibit interactions of its monomers with dentin. Photoactivation timing did not affect the mechanical properties of the two conventional resin cements [39]. In addition, according to Borges et al., when conventional resin cements are used, posts should not be cut immediately after cementation. They used two different resin cements, one conventional combined with an etch-and-rinse adhesive system and one self-adhesive. The posts were cut before cementation, immediately after cementation, or after the restoration of the core. The bond strength values were significantly lower when the posts, which had been cemented with the conventional resin cement, were cut immediately [40]. Silva et al. examined whether magnification, during post space preparation and cleaning, affects the amount of remnant residues and, consequently, the bond strength. They used a self-adhesive resin cement for the cementation of posts, and they divided the samples into three groups according to the method of magnification used: naked eye, a loupe (3× magnification), and a surgical microscope (6× magnification). The bond strength values of the three groups were similar. A micro-CT analysis also showed that there was no difference in the amount of remnant residues between the three groups [41].

## 4. Discussion

The present narrative review aimed to evaluate the factors influencing the adhesive cementation of glass fiber posts.

As was previously mentioned by other authors, the composition of the endodontic sealer plays a critical role and it can negatively affect proper adhesive cementation [7,42]. Eugenol-based sealers must be avoided since they decrease the degree of conversion of the resin cement and negatively affect adhesive bonding [42]. Epoxy resin-based sealers seem to have the best performance when the restoration of the endodontically treated tooth includes a glass fiber post cemented with resin cement [13,14]. The length of the glass fiber post does not seem to play a crucial role in bond strength above a certain length. Even a glass fiber post length equal to 5 mm can show optimal performance in terms of retention and fracture resistance, and it is the proper adaptation of an adhesively cemented post inside the root canal space which is more important [17,18,19].

Many cleaning protocols for the root canal space have already been suggested, and some new, more complex methods including antioxidants and laser irradiation have been developed, with promising results [21,24,43]. It appears that the use of NaOCl and EDTA followed by chlorhexidine leads to acceptable performance concerning the proper adhesive cementation of a glass fiber post [20]. Regarding post surface pretreatment, the results of the relevant studies are inconclusive. Even though some studies support that sandblasting combined with silane application leads to favorable results concerning bond strength [29,30,44], it is possible that sandblasting alone compromises the post’s mechanical properties and reduces bond strength [46]. Silane application seems to be effective especially if it is combined with some sort of etching of the post surface through chemical or mechanical means [25,30]. Furthermore, silane seems to exhibit better performance when it is heated [26,28].

Regarding resin cement type, self-adhesive resin cements are adequate for appropriate glass fiber post adhesive cementation and their management is less technically sensitive compared to conventional resin cements [8,35,47]. During the cementation of the post, resin cement must be applied inside the root canal with an elongation tip to avoid the formation of voids and bubbles [38]. Delayed photoactivation favors adhesive bonding and enhances bond strength regardless of the type of resin cement used [39].

Some of the studies included in this narrative review may be rather old, but there are no more recent studies investigating the factors that affect adhesive bonding between glass fiber posts and root canals. Moreover, each study included in the present review used different methodologies to evaluate bond strength, making comparisons between them difficult. All the studies included in this narrative review are in vitro studies and the systematic reviews discussed are also based on in vitro studies. This is a considerable limitation as the results cannot be directly related to clinical practice. Therefore, evidence coming from long-term randomized prospective clinical trials is needed in order to obtain safer conclusions.

## 5. Conclusions

Based on the articles included in the present review, clinicians should pay attention to some critical steps to achieve the optimal adhesive cementation of glass fiber posts in the root canal. Clinicians should know the type of endodontic sealer used for the root canal obturation and whether it affects the resin cement’s performance. Epoxy resin-based endodontic sealers are recommended and the use of temporary cement in the root canal should be avoided. After post space preparation, the root canal must be adequately cleaned with the appropriate chemical and mechanical means before bonding the post. Irrigating the root canals with chlorhexidine or MTAD or EDTA (alone or in combination with NaOCl) after post space preparation seems to enhance the bond strength. A length of glass fiber posts over 5 mm is adequate in the presence of ferrule. Silane application seems to be beneficial for bond strength, especially when heated, while the role of air abrasion remains controversial. Conventional resin cements in combination with etch-and-rinse adhesive systems or self-adhesive resin cements show the best results so far; however, the literature remains inconclusive on this topic. In any case, resin cements and adhesive systems should be used appropriately according to their limitations and manufacturer’s instructions. Finally, resin cements should be applied inside the root canal with an elongation tip and photoactivation should be delayed.

## Figures and Tables

**Table 1:** Included laboratory studies after screening and selection process.

Authors	Year	Method	Results
Ana P Farina, Emanuele de Oliveira, Alana Disarz, Ana LC de Moura, Migueli Durigon, Matheus A Souza, Doglas Cecchin [12].	2019	60 bovine teeth obturated with eugenol-based sealer and gutta-percha points: 1. negative control (unfilled), 2. positive control (saline solution), 3. 95% alcohol, 4. amylacetate, 5. largo bur.	Cleaning protocols 3 and 5 led to the same bond strength values as the negative control group.
Danielle Araújo Vilas-Boas, Renata Grazziotin-Soares, Diego Machado Ardenghi, José Bauer, Patrícia Oliveira de Souza, George Táccio de Miranda Candeiro, Etevaldo Matos Maia-Filho, Ceci Nunes Carvalho [13].	2018	72 premolars obturated with 1. epoxy-amine resin-based sealer, 2. eugenol-based sealer, 3. bioceramic sealer and gutta-percha points. Posts were cemented either immediately after obturation or seven days after. Bond strength was compared with a control group where no obturation was performed.	Bond strength values of posts cemented at root canals obturated with epoxy-amine resin-based sealer was equal to that of the control group. Eugenol-based and bioceramic sealers showed reduced bond strength values.
Isadora Mello Vilarinho Soares, Bruno Monguilhott Crozeta, Rodrigo Dantas Pereira, Ricardo Gariba Silva, Antonio Miranda da Cruz-Filho [14].	2020	72 canines were divided into 6 groups according to the endodontic sealer used. 1: control; 2: eugenol-based sealer; 3 salicylate-based sealer with calcium hydroxide; 4: epoxy resin-based sealer; 5: epoxy resin-based sealer with calcium hydroxide; and 6: calcium silicate-based sealer.	Group 4 showed the highest bond strength values, similar to the control group.
Joissi Ferrari Zaniboni, Aryvelto Miranda Silva, Cristiane de Melo Alencar, Eduardo Fernandez, Jéssika Mayhara Pereira Morais, Edson Alves de Campos, Milton Carlos Kuga [15].	2021	Temporary metal posts were cemented to 60 root canals using 3 different temporary cements, one eugenol-based and 2 eugenol-free, before glass fiber post cementation with resin cement. Twenty glass fiber posts were cemented to root canals in which no metal post was cemented (control group).	Temporary resin cement negatively affects bond strength values of glass fiber posts regardless of its composition and root canal third.
Mariana Benedetti Ferreira Webber, Silvia Masae de Araújo Michida, Fabiano Carlos Marso, Giovani Corrêa de Oliveira, Cleverson de Oliveira e Silva [16].	2014	Specimens were divided into three groups according to length of the glass fiber posts. Group 1: 2/3 of root canal’s length (14 mm); Group 2: 1/2 (10.5 mm); Group 3: 1/4 (5.25 mm).	Bond strength values did not differ between Group 1 and Group 2.
Shurooq S. Abdulrazzak, Eshamsul Sulaiman, Basim K. Atiya, Marhazlinda Jamaludin [17].	2014	90 maxillary central incisors received glass fiber posts, resin composite core build-up, and cast metal crowns. Specimens were divided into three groups. Group 1: 4 mm ferrule height; Group 2: 2 mm ferrule height; Group 3: 0 mm ferrule height. Each group was further subdivided into 3 groups. Subgroup 1: posts with length equal to 2/3 of root length; Subgroup 2: posts with length equal to 1/2 of root length; Subgroup 3: posts with length equal to 1/3 of root length.	There were significant differences in the failure load in the ferrule height groups, but no significant differences in post length groups and no significant interaction between ferrule heights and post lengths.
Érico Braga Franco, Accacio Linsdo Valle, Ana Lúcia Pompéia Fragade Almeida, José Henrique Rubo, Jefferson Ricardo Pereira [18].	2014	40 canines were divided into 4 groups according to the core build up restoration. Group 1: custom metal post and core-control group; Group 2: glass fiber post with length equal to 1/3 of root’s length (5 mm); Group 3: 1/2 (7.5 mm); Group 4: 2/3 (10 mm).	Control group presented three times higher resistance to static load than the other groups. There were no differences between Groups 2, 3, and 4.
Thiago Lopes de Freitas, Rafael Pino Vitti, Milton Edson Miranda, William Cunha Brandt [19].	2019	40 human teeth with same post space size (1.8 mm cervical, 1.05 mm apical) received glass fiber posts with 4 different sizes. Group 1: 1.6 mm coronal, 0.65 mm apical; Group 2: 1.8 mm coronal, 1.05 mm apical; Group 3: 1.4 mm coronal, 0.65 mm apical; Group 4: customized posts with composite resin.	Group 2 and Group 4 had the highest bond strength values that were significant different from the two other groups.
M. Durski, M. Metz, G. Crim, S. Hass, R. Mazur, S. Vieira [20]	2018	120 premolars were divided into 12 groups according to 1. treatment with chlorhexidine, 2. type of resincement (conventional/self-adhesive), and 3. Number of thermal cycles (0/20,000/40,000).	Additional treatment with chlorhexidine before the cement application produced significantly higher bond strength values regardless of cement, root thirds, or thermocycling.
Beatriz Dansini Cândido, Tatiane Miranda Manzoli, Joissi Ferrari Zaniboni, Joao Felipe Besegato, Eduardo Fernández Godoy, Milton Carlos Kuga, Andréa Abi Rached Dantas [21].	2023	60 bovine roots were divided into 6 groups according to the root canal irrigants used before the cementation of the post. Group 1: 2.5% NaOCl + 17% EDTA; Group 2: 2.5% NaOCl + 17% EDTA + 10% sodium ascorbate; Group 3: 2.5% NaOCl + 17% EDTA + 20% alpha-tocopherol; Group 4: peracetic acid; Group 5: peracetic acid + 10% sodium ascorbate; Group 6: peracetic acid + 20% alpha-tocopherol.	Groups 2, 3, 4, 5, and 6 had similar bond strength values.
Mazen F. Alkahtany [22]	2022	140 premolars were divided into 4 groups according to the root canal irrigant used. Group 1: NaOCl + EDTA; Group 2: NaOCl + MTAD; Group 3: NaOCl + riboflavin; Group 4: NaOCl + pineapple peel extract.	Group 2 and Group 4 showed the highest bond strength values with no significant difference between them.
Hamid Jalali, Farzaneh Farid, Sudabeh Kulivand, Saeed Nokar, Kosar Dadgar [23].	2018	72 premolars were divided into 6 groups according to the root canal irrigant used before the cementation of the post. Group 1: Saline solution control group; Group 2: NaOCl; Group 3: EDTA; Group 4: chlorhexidine; Group 5: MTAD; Group 6: phosphoric acid.	Groups 3, 5, and 6 showed the highest bond strength values, which were similar to each other and significantly different from the control group. The difference between the control group and Group 4 was marginally significant.
Shu Wan, Yujie Tan, Jing Xie, Xiaoyu Huang, Ling Guo [24].	2020	55 human mandibular premolars were divided into 5 groups according to the root canal irrigant or technique used before the cementation of the glass fiber posts. Group 1: control group (distilled water); Group 2: 2.5% NaOCl + 17%EDTA; Group 3: MTAD; Group 4: PIPS technique; Group 5: MTAD + PIPS technique.	Group 5 displayed the best result concerning smear layer removal and bond strength values.
Amr M. Elnaghy, Shaymaa E. Elsaka [25].	2014	75 human teeth received glass fiber posts cemented with dual-cure self-adhesive resin cement. Specimens were divided according to the surface treatment of the posts: Control group 1: no treatment; Group 1: silanization for 60 s; Group 2: airborne-particle abrasion; Group 3: etching with 9% hydrofluoric acid for 1 min; and Group 4: etching with CH_2_ Cl_2_ for 10 min.	Group 4 showed a significantly higher bond strength than the other groups. The bond strength of Group 1 was equal to the bond strength of the control group. The bond strength of Group 2 and Group 3 was significantly lower than the bond strength of the control group.
P. Samimi, V. Mortazavi, F. Salamat [26].	2014	40 human teeth received glass fiber posts. Specimens were divided into 5 groups according to post surface treatment. Group 1: hydrofluoric acid etching and silane application; Group 2: hydrofluoric acid etching and silane application that received heat treatment; Group 3: hydrogen peroxide etching and silane application; Group 4: hydrogen peroxide etching application that received heat treatment; Control group: no pretreatment. Conventional resin cement combined with self-etch adhesive system was used.	Bond strength was significantly affected by the heat treatment of the applied silane.
Chandrakanth Majeti, Chandrasekhar Veeramachaneni, Pradeep Kumar Morisetty, Saggurti Anitha Rao, Muralidhar Tummala [27].	2014	90 human maxillary central incisors received glass fiber posts luted with self-adhesive resin cement. Posts were divided into 3 groups based on the surface treatment they received (phosphoric acid 37%, hydrogen peroxide 24%, and distilled water). Each surface treatment method was applied for 3 different time periods (15 s, 30 s, 60 s).	The highest bond strength values were acquired when phosphoric acid was used for 15 s and when hydrogen peroxide was used for 60 s. There was no significant difference between those two groups.
Bruna Cristinado Nascimento Rechia, Ruth Peggy Bravo, Naylin Danyelede Oliveira Carla Castiglia Gonzaga, Flares Baratto Filho, Carmen L. Mueller Storrer [28].	2016	30 glass fiber posts were cemented to 30 bovine root canals with self-adhesive resin cement. Specimens were divided into 3 groups according to the surface treatment of the posts. Group 1: silane application; Group 2: silane and adhesive system application; Group 3: silane application dried with hot air.	Group 2 and Group 3 had the highest bond strength values with no significant difference between them.
Lairds Rodrigues dos Santos, Darlon Martins Lima, Edilausson Moreno Carvalho, Vandilson Pinheiro Rodrigues, Claudia Maria Coelho Alves [29].	2021	84 bovine teeth received glass fiber posts. The specimens were divided into 6 groups according to the post’s surface pretreatment. Group 1: no surface pretreatment; Group 2: silane application; Group 3: 24% hydrogen peroxide application; Group 4: 24% hydrogen peroxide and silane application; Group 5: air abrasion (50 μm aluminum oxide particles); Group 6: air abrasion and silane application.	Groups 2, 4, and 6 had the highest bond strength values with no significant difference between them.
Sriparna Jana, Priti D. Desai, Debojyoti Das, Paromita Mazumdar, Tushar Kanti Majumdar [30].	2022	40 maxillary central incisors received glass fiber posts. The specimens were divided into 4 groups according to the post’s surface pretreatment. Group 1: silane application; Group 2: air abrasion (30 μm silicone dioxide particles) and silane application; Group 3: 9% hydrofluoric acid and silane application; Group 4: silane and universal bond application.	Group 2 had the highest mean bond strength values.
Karla G. F. Gomes, Natália S. Faria, Walter R. Neto, Vivian Colucci, Erica A. Gomes [31].	2017	32 bovine teeth received glass fiber posts. The specimens were divided into 4 groups according to the surface pretreatment of the posts. Group 1: silane application control group; Group 2: irradiation with Er:YAG laser; Group 3: irradiation with Er;Cr:YSSG laser; Group 4: irradiation with 980 nm diode laser.	Group 3 had the highest mean bond strength values.
Khuthija Khanum HM, Ali Abdulmajeed Barakat, Zeeshan Qamar, R. Naveen Reddy, Swetha Vempalli, Anas Hussam Ramadan, Fayez Niazi, Mohammed Noushad [32]	2022	50 premolars received glass fiber posts. The specimens were divided into 5 groups according to the post surface disinfection protocol. Group 1: autoclave sterilization; Group 2: chlorhexidine digluconate; Group 3: CPS solution; Group 4: 35% phosphoric acid gel; Group 5: no disinfection.	Group 2 and Group 4 had the highest bond strength values that were significantly different from the other groups.
Camilo Andrés Pulido, Ana Paula Gebertde Oliveira Franco, Giovana Mongruel Gomes, Bruna Fortes Bittencourt, Hypolito José Kalinowski, João Carlos Gomes, Osnara Maria Mongruel Gomes [10].	2016	30 maxillary canines received glass fiber posts. Specimens were divided into 2 groups according to the cementation system. Group 1: conventional resin cement combined with etch-and-rinse adhesive system; Group 2: self-adhesive resin cement.	Group 1 and Group 2 had similar polymerization shrinkage values. Group 1 had higher degree of conversion values. Bond strength was significantly higher in the cervical region only when conventional resin cement was used.
Guido Migliau, Luca Piccoli, Stefano Di Carlo, Giorgio Pompa, Laith Konstantinos Besharat, Marco Dolci [33].	2017	30 teeth divided into 3 groups according to the resin cement/adhesive system used for the adhesive luting of the posts. Group 1: Conventional resin cement combined with total etch adhesive system; Group 2: self-adhesive resin cement; Group 3: conventional resin cement combined with self-etch adhesive system.	Group 1 had double mean bond strength values than Groups 2 and 3.
Ana Michelle Oliveira Nadler, Evair Josino da Silva, Paulo Cardoso Lins Filho, Marlon Ferreira Dias, Renata Pedrosa Guimarães, Claudio Heliomar Vicente da Silva, Sérgiodos Santos Silva, Anderson Stevens Leonidas Gomes [34].	2023	55 premolars divided into 5 groups according to the resin cement/adhesive system used for the adhesive luting of the posts. Groups 1, 2, and 3: conventional resin cement combined with etch-and-rinse adhesive system; Group 4: conventional resin cement combined with self-etch adhesive system; Group 5: self-adhesive resin cement.	Groups 2 and 3 had the highest mean bond strength values. Group 3 had similar bond strength values as Group 4 and Group 5 in the middle and in the apical third of the root canal. Group 1 had similar bond strength values with Group 4 and Group 5 in the cervical and the middle third of the root canal.
Batu Can Yaman, Fusun Ozer, Takuro Takeichi, Bekir Karabucak, Fatma Koray, Markus B. Blatz [35].	2014	Glass fiber posts or zirconia posts were luted to root canals of 80 premolars. Specimens were divided into 2 main groups according to the resin cement/adhesive system used. Group 1: conventional resin cement combined with self-etch adhesive system; Group 2: self-adhesive cement. In each group, half of the specimens were subjected to a thermomechanical aging procedure.	For glass fiber posts without TMA, Group 1 had higher bond strength values than Group 2. After the TMA bond strength values of Group 1 were reduced, Group 2 had higher bond strength values than Group 1.
Daphne Câmara Barcellos, Maria Filomena Rochalima Huhtala, Melissa Aline Silva, Ana Paula Martins Gomes, Lucas Teixeira Franco [36].	2014	Glass fiber posts were luted to root canals of 25 human teeth using the same conventional resin cement in combination with a. a self-etch adhesive system; b. etch-and-rinse adhesive system (etching with phosphoric acid 37% for 15 s.)	Bond strength values, when self-etch adhesive system was used, were double that of those acquired when the etch-and-rinse adhesive system was used.
Eluise C. Rezende, Giovana Mongruel Gomes, Anna Luiza Szesz, Carlos Eduardo da Silveira Bueno, Alessandra Reise, Alessandro D. Loguercio [37].	2016	72 glass fiber posts were luted to premolars. Specimens were divided into 6 groups according to the combination of 2 factors: resin cement/adhesive system (2 different etch-and-rinse adhesive systems and 2 different conventional resin cements) and dentine moisture (dry, wet, and overwet).	For both adhesive systems/resin cements, higher bond strength values and lower nanoleakages were observed when the dentin was wet, and lower bond strength values and higher nanoleakage were observed when the dentin was dry. The results when the dentin was overwet were intermediate.
MT. Durski, MJ. Metz, JY. Thompson, AK. Mascarenhas, GA. Crim, S. Vieira, R. Mazur [38].	2016	60 glass fiber posts were luted to premolars. Specimens where divided into 3 groups according to the resin cement. Group 1: conventional resin cement in combination with etch-and-rinse adhesive system; Group 2: self-adhesive resin cement; Group 3: the same self-adhesive resin cement in combination with an extra step of phosphoric acid etching. Each group further divided into 3 subgroups according to cement application technique. Subgroup 1: application with an elongation tip; Subgroup 2: application with a microbrush.	Higher mean bond strength values at the apical third of all groups were observed when the resin cement was applied with the elongation tip compared to the micro brush. Resin cement application with the elongation tip led to higher bond strength values at the cervical and the middle thirds for Group 2. The extra etching step before the self-etch adhesive cementation appeared to be effective in enhancing bond strength.
RD. Pereira, Valdivia, AA. Bicalho, SD. Franco, D. Tantbirojn, A. Versluis, CJ. Soares [39].	2015	135 glass fiber posts were luted to bovine teeth with 3 different resin cements. Group 1: self-adhesive resin cement; Group 2: conventional resin cement in combination with self-etch adhesive system; Group 3: conventional resin cement in combination with etch-and-rinse adhesive system. Three different photoactivation timings were used: 1. light curing immediately; 2. after 3 min; 3. after 5 min.	Higher bond strength values were achieved when photoactivation timing 2 and 3 were used in all groups, with significant differences between photoactivation timing 2 and photoactivation timing 3 for Group 1 and Group 3. Photoactivation timing affected the resin cement’s mechanical properties in Group 1. Shrinkage stress values decreased gradually with delayed photoactivation in all groups.
Marcela G. Borges, André L. Faria-e-Silva, Paulo C. F. Santos-Filho, Fernanda P. Silva, Luís R. M. Martins, Murilo de Sousa Menezes [40].	2015	60 glass fiber posts were cemented to bovine incisors using 1. conventional resin cement in combination with etch-and-rinse adhesive system and 2. self-adhesive resin cement. Posts were cut 1. prior to post cementation; 2. immediately after post cementation; 3. after core build up.	Bond strength was reduced when conventional resin cement was used and the post was cut immediately.
Natércia Rezende da Silva, Monise de Paula Rodrigues, Aline Aredes Bicalho, Raissa Albuquerque de Deus1 Soares, Carlos José Soares, Priscilla Barbosa Ferreira [41].	2019	30 central upper incisors were divided into 3 groups according to magnification method used during the cementation process. Group 1: naked eye; Group 2: 3× magnification using dental loupe; Group 3: 6× magnification using microscope.	No difference in the amount of residues and in bond strength values were observed between the 3 groups.
